# The effects of B‐cell–activating factor on the population size, maturation and function of murine natural killer cells

**DOI:** 10.1111/imcb.12585

**Published:** 2022-10-08

**Authors:** Pin Shie Quah, Vivien Sutton, Eden Whitlock, William A Figgett, Daniel M Andrews, Kirsten A Fairfax, Fabienne Mackay

**Affiliations:** ^1^ Department of Immunology and Pathology Central Clinical School, Monash University Melbourne VIC Australia; ^2^ Department of Microbiology and Immunology The University of Melbourne, Peter Doherty Institute for Infection and Immunity Melbourne VIC Australia; ^3^ Rosie Lew Cancer Immunology Program Peter MacCallum Cancer Centre Melbourne VIC Australia; ^4^ Sir Peter MacCallum Department of Oncology The University of Melbourne Melbourne VIC Australia; ^5^ QIMR Berghofer Medical Research Institute Herston QLD Australia; ^6^ Garvan Institute of Medical Research Darlinghurst NSW Australia; ^7^ Bioproperties, Ringwood Melbourne VIC Australia; ^8^ Blood Cells and Blood Cancer Division The Walter and Eliza Hall Institute of Medical Research Parkville VIC Australia; ^9^ Menzies Institute for Medical Research University of Tasmania Hobart TAS Australia; ^10^ School of Medicine, College of Health and Medicine University of Tasmania Hobart TAS Australia; ^11^ Faculty of Medicine The University of Queensland Brisbane QLD Australia

**Keywords:** Autoimmunity, Cancer, Immunodeficiency, Innate immunity, Natural killer cell

## Abstract

The role of B‐cell–activating factor (BAFF) in B‐lymphocyte biology has been comprehensively studied, but its contributions to innate immunity remain unclear. Natural killer (NK) cells form the first line of defense against viruses and tumors, and have been shown to be defective in patients with systemic lupus erythematosus (SLE). The link between BAFF and NK cells in the development and progression of SLE remains unstudied. By assessing NK cell numbers in wild‐type (WT), BAFF^−/−^ (BAFF deficient), BAFF‐R^−/−^ (BAFF receptor deficient), TACI^−/−^ (transmembrane activator and calcium modulator and cyclophilin ligand interactor deficient), BCMA^−/−^ (B‐cell maturation antigen deficient) and BAFF transgenic (Tg) mice, we observed that BAFF signaling through BAFF‐R was essential for sustaining NK cell numbers in the spleen. However, according to the cell surface expression of CD27 and CD11b on NK cells, we found that BAFF was dispensable for NK cell maturation. *Ex vivo* and *in vivo* models showed that NK cells from BAFF^−/−^ and BAFF Tg mice produced interferon‐γ and killed tumor cells at a level similar to that in WT mice. Finally, we established that NK cells do not express receptors that interact with BAFF in the steady state or in the BAFF Tg mouse model of SLE. Our findings demonstrate that BAFF has an indirect effect on NK cell homeostasis and no effect on NK cell function.

## Graphical abstract

This study is the first detailed investigation of the participation of B‐cell–activating factor (BAFF) of the tumour necrosis factor family in natural killer (NK) cell number and function. In this paper, we show that BAFF has an indirect effect on NK cell numbers but has no effect on their functions by utilising *ex vivo* and *in vivo* techniques.

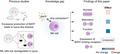

## INTRODUCTION

The cytokine known as B‐cell–activating factor (BAFF; TNFSF13B) is a ligand in the tumor necrosis factor ligand superfamily.^1^ The receptors for BAFF, BAFF receptor (BAFF‐R; TNFRSF13C), transmembrane activator and calcium modulator and cyclophilin ligand interactor (TACI; TNFRSF13B) and B‐cell maturation antigen (BCMA; TNFRSF17), are expressed predominantly by B cells and antibody‐producing plasma cells. BAFF‐R binds exclusively to BAFF, whereas TACI and BCMA can bind to BAFF and its homolog, a proliferation‐inducing ligand (APRIL; TNFSF13).^1^ BAFF‐R and BCMA signaling trigger survival signals in peripheral B cells and long‐lived bone marrow (BM) plasma cells, respectively.^2^, ^3^ Survival signals in plasma cells are also generated when high‐order BAFF and APRIL oligomers bind to TACI.^4^ Interestingly, TACI plays a regulatory role in innate‐like B cells, such as marginal zone B cells, by triggering activation‐induced cell death.^5^ TACI also supports other functions of B cells, including T‐independent antibody responses, isotype class switching and interleukin (IL)‐10 production.^6^, ^7^, ^8^ BCMA plays additional roles in B‐cell–mediated antigen presentation.^9^ The expression of receptors for BAFF is not limited to B cells, with BAFF‐R expression also noted on activated and memory T‐cell subsets.^10^

Elevated circulating BAFF levels are frequently observed in patients with autoimmune disorders such as systemic lupus erythematosus (SLE), rheumatoid arthritis, Sjögren's syndrome and systemic sclerosis, suggesting that BAFF is a pathogenic factor for autoimmune diseases.^1^, ^11^, ^12^, ^13^ Notably, transgenic (Tg) mice that overexpress BAFF have an expanded B‐cell compartment.^14^ In these mice, high BAFF levels allow low‐affinity self‐reactive B cells to escape tolerance mechanisms, driving lupus‐like disease characterized by high levels of proinflammatory antinuclear autoantibodies, glomerulonephritis (from immunoglobulin deposition in the kidneys) and proteinuria.^14^, ^15^ The discovery that BAFF is involved in SLE led to the development of belimumab, a BAFF‐neutralizing monoclonal antibody. In 2011, belimumab was approved by the Food and Drug Administration as the first targeted biological treatment for SLE.^16^ In 2020, belimumab was further approved by the Food and Drug Administration as the first treatment for lupus nephritis.^17^

Natural killer (NK) cells are large granular lymphocytes of the innate immune system that serve as a vital first line of defense against tumor cells and intracellular pathogens, including viruses and bacteria.^18^ Similar to B and T lymphocytes, NK cells develop in the BM from common lymphoid progenitors.^19^ However, unlike B and T cells, NK cells do not undergo negative selection to acquire self‐tolerance.^18^ NK cells have been implicated in disorders that specifically involve BAFF and BAFF‐mediated signaling,^20^, ^21^, ^22^ but the direct impact of this signaling pathway on NK cell function remains unclear.

In healthy humans and wild‐type (WT) mice, BAFF is produced primarily by innate immune cells, including neutrophils, monocytes, macrophages and dendritic cells.^1^ Gene expression databases, including https://www.haemosphere.org, show that NK cells from humans and WT mice express BAFF transcripts in the steady state, albeit at low levels.^23^ Interestingly, BAFF levels were higher in peripheral blood mononuclear cell–derived NK cells than in monocytes upon stimulation with IL‐2.^24^ NK cells from patients with specific inflammatory diseases may therefore be induced to produce high levels of BAFF.

Shan *et al*.^25^ have reported that intraperitoneal injections of BAFF results in a dose‐dependent enhancement of NK cell activity. Although this finding suggests that NK cells may respond directly to BAFF, this hypothesis was rejected by Zhang *et al*.^26^ who found that the effects of BAFF on NK cell activity are dependent on the actions of T‐cell–derived IL‐2 and interferon (IFN)‐γ. A current gap in the literature, which our study addresses, is whether NK cells express receptors that are capable of binding directly to BAFF.

Functional NK cell defects have been reported in patients diagnosed with SLE.^21^ Moreover, hematopoietic stem cells from patients with SLE displayed less capacity to differentiate into mature NK cells than those from healthy controls.^27^ While the mechanisms underlying the reduced NK cell numbers and their functional deficiencies are not well understood, several NK cell–associated defects in patients with SLE have been characterized. These include reduced IL2Rβ expression, hyporesponsiveness to IL‐15–mediated stimulation and suppression by antilymphocyte antibodies.^21^ Reduced NK cell numbers and activity have also been reported in patients diagnosed with primary Sjögren's syndrome.^28^ Treatment of a cohort of patients with Sjögren's syndrome using belimumab resulted in increased numbers of NK cells in both the blood and salivary glands.^29^ Likewise, while BAFF‐treated chronic lymphocytic leukemia cells exhibited increased resistance to direct NK‐mediated cell death as well as to antibody‐dependent cellular toxicity, *in vitro* studies found that belimumab enhanced NK cell reactivity and antibody‐dependent cellular toxicity in these patients.^20^ The results of these studies collectively suggest that the actions of BAFF may suppress both NK cell numbers and NK cell–mediated cytotoxicity in the context of disease.

The impact of BAFF on NK cell homeostasis has not been studied in patients diagnosed with immunodeficiency disorders. Two patients with common variable immunodefiency (CVID) associated with homozygous deletions within the gene encoding BAFF‐R have so far been reported.^22^ These patients exhibited reduced percentages and numbers per microliter of circulating B cells. In one of the patients, the percentage and number of NK cells were also reduced while the distribution of T cells remained within the normal range.^22^ It is not clear whether the NK cell deficiency seen in this patient resulted directly from the absence of BAFF‐R expression, or indirectly by substantially altering the cellular environment with a loss of mature B cells. This question is difficult to address using human samples because only 2 out of 138 patients with CVID (0.15%) exhibited a complete absence of BAFF‐R expression,^22^ but it can be explored using BAFF‐deficient (BAFF‐R^−/−^) mice. Similarly, TACI‐deficient (TACI^−/−^) mice can be used to study the role of TACI in NK cell homeostasis. The function of NK cells from humans who carry homozygous *TACI* mutations has not be investigated.^30^

As excessive BAFF production leads to lupus‐like disease,^14^ and NK cells have been shown to play a role in lupus,^21^ this study explored a previously uncharacterized area, investigating the role of BAFF and its receptors in NK cell biology, and the impact of dysregulated BAFF on NK cell homeostasis and function. As no studies have described the direct effect of BAFF on NK cells *via* BAFF‐R signaling, we also assessed whether NK cells might express BAFF‐binding receptors. We found that BAFF has an indirect effect on NK cell homeostasis but no effect on NK cell function.

## RESULTS

### Impact of BAFF and its receptors on the size of NK cell populations in the liver, bone marrow and spleen

**Figure 1:**
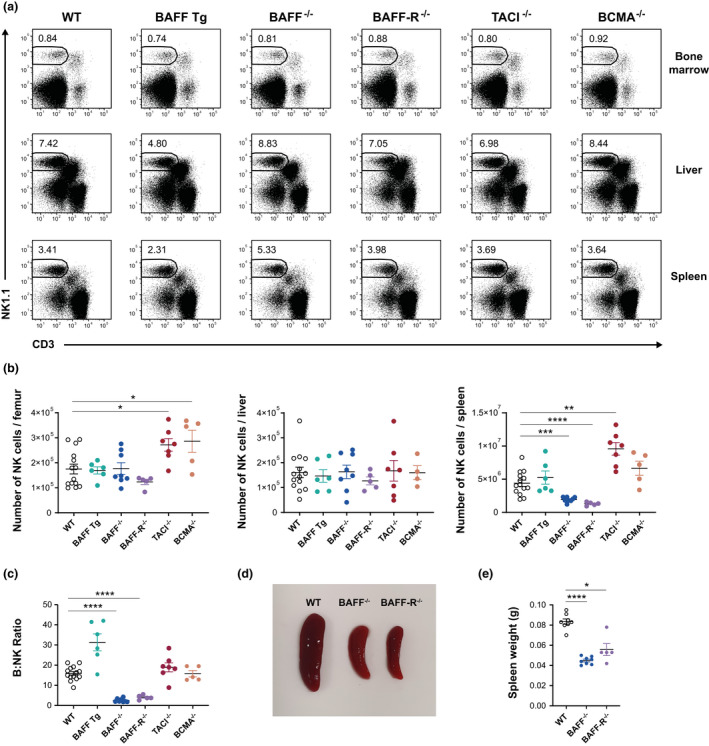
Phenotypic analysis of NK cells in the BM, liver and spleen of BAFF Tg, BAFF^−/−^, BAFF‐R^−/−^, TACI^−/−^ and BCMA^−/−^ mice compared with WT controls. The NK population was identified as CD19^−^CD3^−^NK1.1^+^ cells. **(a)** Representative plots showing the mean percentages of live NK cells in WT and GM mice at three anatomical locations. **(b)** Absolute numbers of NK cells in the BM, liver and spleen of WT and GM mice. **(c)** The ratio of B to NK cells in each spleen, calculated as the number of B cells divided by the number of NK cells. **(d)** Relative size and **(e)** weight of spleens isolated from WT, BAFF^−/−^ and BAFF‐R^−/−^ mice. Data are shown as means ± standard error of the mean, and each symbol represents the results from a single mouse. All data are representative of two or three independent experiments; *n* = 4–14 mice; *P ≤ 0.05, **P ≤ 0.01, ***P ≤ 0.001, ****P ≤ 0.0001. BAFF, B‐cell–activating factor; BAFF‐R, B‐cell–activating factor receptor; BCMA, B‐cell maturation antigen; BM, bone marrow; GM, genetically modified; NK, natural killer (cells); TACI, transmembrane activator and calcium modulator and cyclophilin ligand interactor; Tg, transgenic; WT, wild type.

Previous studies have reported correlations between NK cell activity and BAFF,^25^, ^26^ and demonstrated that NK cells produce large amounts of BAFF when stimulated.^24^ These results suggest the existence of a link between BAFF activity and NK cells, but to our knowledge, comprehensive analysis of the impact of BAFF and any of its receptors on NK cell homeostasis remains unavailable. To evaluate the effect of BAFF‐mediated signaling on NK cell numbers, we examined tissues from animal models with enhanced or restricted BAFF signaling, namely, heterozygous BAFF Tg, BAFF^−/−^, BAFF‐R^−/−^, TACI^−/−^ and BCMA^−/−^ mice (Supplementary table 1). Age‐ and sex‐matched WT C57BL/6J mice were used as the controls for these genetically modified (GM) mice. As the development and maturation of NK cells is niche dependent, we examined NK cell populations in several organs, including the BM, liver and spleen. The gating strategy used to identify NK cells is shown in Supplementary figure 1. Representative flow cytometry plots documenting the percentage of NK cells (CD3^−^NK1.1^+^) in different organs of each mouse strain are shown in Figure 1a.

Our results demonstrate that the BAFF system has no effect on the size of NK cell populations in the liver (Figure 1b). While the numbers of BM NK cells in BAFF Tg, BAFF^−/−^ and BAFF‐R^−/−^ mice were comparable to those in WT mice, the numbers of BM NK cells increased by 1.6‐fold in both TACI^−/−^ (*P* = 0.0161) and BCMA^−/−^ (*P* = 0.0133) mice (Figure 1b). This suggests that BAFF signaling through BAFF‐R is not required to control NK cell numbers, but that signals downstream of TACI and BCMA, either extrinsic or intrinsic, may play a role in restricting NK cell populations in the BM. Of note, both TACI and BCMA bind to APRIL in addition to BAFF.

Overexpression of BAFF and deletion of BCMA had minimal to no statistically significant change in the size of the splenic NK cell population. As in the BM, TACI deletion led to a significant increase in splenic NK cell numbers (Figure 1b). Deletion of BAFF and BAFF‐R led to a significant reduction in splenic NK cell numbers; the number of splenic NK cells was reduced by a factor of 2.3 (*P* ≤ 0.001) in BAFF^−/−^ mice and 3.4 (*P* ≤ 0.0001) in BAFF‐R^−/−^ mice (Figure 1b). We note that the differences in NK cell frequencies observed in BAFF^−/−^ and BAFF‐R^−/−^ mice reflected an observed underrepresentation of B cells in the spleens of these mice (Figure 1c). Given that BAFF‐R^−/−^ and BAFF^−/−^ mice have smaller spleens (Figure 1d, e), we do not exclude that an altered splenic architecture, as a result of the lack of mature B cells in BAFF^−/−^ and BAFF‐R^−/−^ mice, may have affected NK cell numbers.

### Impact of BAFF and its receptors on NK cell maturation in the bone marrow and spleen

**Figure 2:**
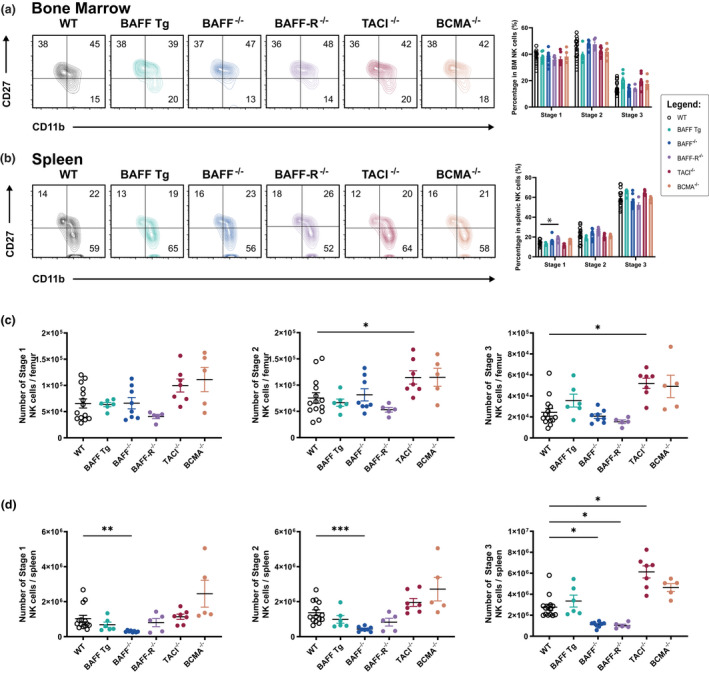
The distribution and population sizes of NK cell subsets including Stage 1 (CD11b^low^CD27^high^), Stage 2 (CD11b^high^CD27^high^) and terminally differentiated Stage 3 cells (CD11b^high^CD27^low^). The distribution of NK cell subsets in the **(a)** BM and **(b)** spleens of WT and GM mice. The numbers within the FACS plots in **a** and **b** are the mean percentages of each CD3^−^NK1.1^+^ population as a function of the total NK cell population. Absolute numbers (means ± standard error of the mean) of cells of each NK subset in the **(c)** bone marrow and **(d)** spleen. Each symbol represents the findings from one mouse. The GM mice were age and gender matched to the WT controls. All data shown are representative of at least two independent experiments with *n* = 5–14 mice per strain; *P ≤ 0.05, **P ≤ 0.01, ***P ≤ 0.001. BAFF, B‐cell–activating factor; BAFF‐R, B‐cell–activating factor receptor; BCMA, B‐cell maturation antigen; BM, bone marrow; FACS, fluorescence‐activated cell sorting; GM, genetically modified; NK, natural killer (cells); TACI, transmembrane activator and calcium modulator and cyclophilin ligand interactor; Tg, transgenic; WT, wild type.

Mouse NK cells can be classified into four subsets along a progression of maturity based on their expression of the cell surface antigens CD27 and CD11b. The most immature NK cells (CD27^low^CD11b^low^) differentiate in sequence into cells that are CD27^hi^CD11b^low^ (stage 1), CD27^hi^CD11b^hi^ (stage 2) and CD27^low^CD11b^hi^ (stage 3).^31^ Given that our results link BAFF‐mediated signaling to NK cell numbers, we investigated the impact of BAFF on NK cell maturation status and found that the maturation status of BM NK cells remained unchanged in BAFF Tg, BAFF^−/−^, BAFF‐R^−/−^, TACI^−/−^ and BCMA^−/−^ mice compared with the WT mice (Figure 2a). There was a slight elevation in the frequency of stage 1 NK cells (*P* ≤ 0.05) in the spleens of BAFF‐R^−/−^ mice (Figure 2b), suggesting that BAFF‐R deletion mildly skewed splenic NK cells toward the more immature phenotype. However, because of the small spleen size in BAFF‐R^−/−^ mice, this effect was not robust enough to lead to a statistically significant numerical increase in BAFF‐R^−/−^ stage 1 splenic NK cells (Figure 2d).

The absolute numbers of BM stage 2 (*P* ≤ 0.05) and stage 3 (*P* ≤ 0.05) NK cells were higher in TACI^−/−^ compared with WT controls (Figure 2c), but the percentages of each of the maturation stages was similar to those in the WT controls (Figure 2a), indicating that there was no specific maturation difference, but rather an increase in total numbers of NK cells. In the spleen, the absolute numbers of stage 1 (*P* ≤ 0.01), stage 2 (*P* ≤ 0.001) and stage 3 (*P* ≤ 0.05) were reduced in BAFF^−/−^ mice (Figure 2d), but again the percentages of each maturation stage were similar to those of the WT controls (Figure 2b), indicating that there was no specific maturation defect. In the spleens of BAFF‐R^−/−^ mice, the number of stage 3 NK cells (*P* ≤ 0.05) was lower (Figure 2d), whereas the number of stage 3 splenic NK cells in TACI^−/−^ mice (*P* ≤ 0.05) was greater than that of the WT controls (Figure 2d); again, it should be noted that differences in the numbers of NK cell subsets reflect the aforementioned differences in total cell numbers (Figure 1b). Overall, we did not observe any impact of the BAFF system on NK cell maturation. The variations observed in NK cell numbers were consistent with the variations in spleen sizes.

### BAFF has no impact on NK cell function

Previous reports have documented a positive correlation between BAFF levels and NK cell cytotoxicity.^25^, ^26^ However, the direct effect of BAFF on NK cells was never tested. Here, we employed both *ex vivo* and *in vivo* assays to evaluate the consequences of BAFF‐mediated signaling on NK cell function.

**Figure 3:**
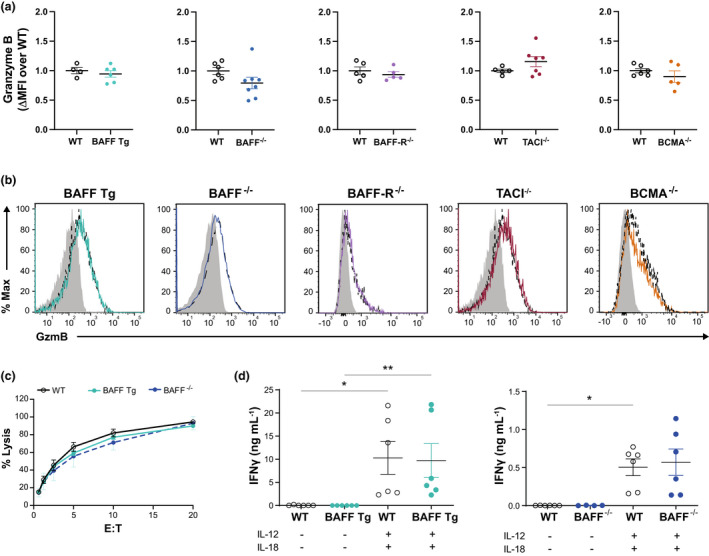
Analysis of NK cell function *ex vivo*. **(a)** ΔMFI of GzmB = geometric MFI of the CD3^−^NK1.1^+^ population – geometric MFI of the CD3^−^NK1.1^−^ population. As the data were pooled from two or three independent experiments, the ΔMFI of each mouse was normalized as the fold‐change over the average ΔMFI of the WT mice in each experiment. **(b)** Histograms documenting the expression of GzmB in the CD3^−^NK1.1^+^ population from GM mice (solid line) compared with WT controls (dashed line). The shaded histograms document GzmB expression in the negative control (CD3^−^NK1.1^−^) cell population. **(c)** ^51^Cr release assay performed on resting NK cells isolated from the spleens of WT, BAFF transgenic (Tg) and BAFF^−/−^ mice at various E:T ratios. **(d)** NK cells (CD3^−^NK1.1^+^) were isolated at over 95% purity and cultured at 10^6^ cells mL^−1^ with or without IL‐12 (1 ng mL^−1^) and IL‐18 (5 ng mL^−1^). Supernatants were collected at *t* = 6 h and analyzed using a LEGENDplex cytometric bead array to detect IFNγ. The data (mean ± standard error of the mean) presented in **a** (*n* = 5–14 mice) and **d** (*n* = 4–6 mice) are pooled from two or three independent experiments. The data presented in **b** (*n* = 5–14 mice) and **c** (*n* = 4 mice) are representative of two or three independent experiments. *P ≤ 0.05, **P ≤ 0.01. BAFF, B‐cell–activating factor; BAFF‐R, B‐cell–activating factor receptor; BCMA, B‐cell maturation antigen; E:T, effector‐to‐target ratio; GM, genetically modified; GzmB, granzyme B; IFN, interferon; IL, interleukin; MFI, mean fluorescence intensity; NK, natural killer (cells); TACI, transmembrane activator and calcium modulator and cyclophilin ligand interactor; WT, wild type.

NK cells kill pathogen‐infected and tumor cells by synthesizing and releasing granzymes. Of the 10 distinct granzymes that have been identified in mice, granzyme B has the most potent proapoptotic activity.^32^ We therefore evaluated the expression of granzyme B in the splenic NK cells of WT, BAFF Tg, BAFF^−/−^, BAFF‐R^−/−^, TACI^−/−^ and BCMA^−/−^ mice. Granzyme B expression in NK cells from all the GM mouse strains used in our study showed no statistically significant differences to that detected in the WT controls (Figure 3a, b). These results suggest that disruption of BAFF‐mediated signaling pathways has no or limited impact on NK cell cytotoxicity.

To confirm this hypothesis, we performed a chromium release assay with NK cells isolated from the spleens of BAFF^−/−^ and BAFF Tg mice. NK cells from these GM mice were as effective at target cell lysis as NK cells from WT mice (Figure 3c). NK cells are also a primary source of IFNγ, a cytokine that is critical for combating tumor growth and infection with intracellular pathogens. The cytokines IL‐12 and IL‐18 act synergistically to induce high levels of IFNγ production in NK cells.^33^ To examine whether BAFF‐mediated signaling is required for IFNγ production and to test whether prior exposure to chronically high or low levels of BAFF affects IFNγ production, we isolated splenic NK cells from WT, BAFF^−/−^ and BAFF Tg mice and cultured the cells for 6 h with IL‐12 and IL‐18. We observed no differences in IFNγ production, as IL‐12– and IL‐18–stimulated NK cells from the WT, BAFF Tg and BAFF^−/−^ mice released higher amounts of IFNγ than their unstimulated controls in a similar way (Figure 3d). These results suggest that BAFF‐mediated signaling is not required for IFNγ production by activated NK cells. Overall, our findings provide no evidence that BAFF has any major effect on NK cell function *in vitro*.

**Figure 4:**
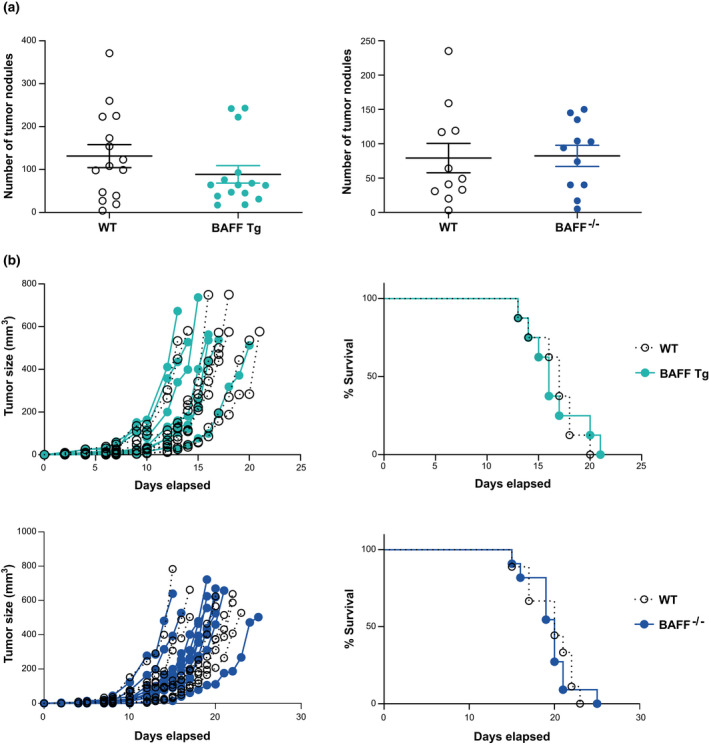
Analysis of NK cell function *in vivo*. **(a)** The metastatic burden of B16‐F10 melanoma cells in the lungs of BAFF Tg and BAFF^−/−^ mice compared with WT controls. **(b)** Tumor growth and survival of B16‐F1 melanoma‐bearing mice. Data shown in **a** are means ± standard error of the mean from 11 and 15 mice pooled from two or three independent experiments. Data shown in **b** are from 15–18 mice and are representative of two independent experiments. BAFF, B‐cell–activating factor; NK, natural killer (cells) Tg, transgenic; WT, wild type.

As an alternative approach, we utilized syngeneic B16 melanoma implantation models to examine NK cell function *in vivo*.^34^ In this study, we used B16‐F10 cells in an experimental tumor metastasis model in which cells introduced intravenously would migrate and accumulate in the lungs.^34^ The control of tumor growth in this model is dependent on NK cells, and independent from cytotoxic CD8^+^ T cells.^35^ This is unsurprising, as B16 melanoma cells express low levels of major histocompatibility complex class I.^36^ Our results show no statistically significant differences in the number of tumor nodules detected on the surfaces of lungs of BAFF^−/−^ or BAFF Tg mice *versus* the WT controls (Figure 4a), demonstrating that BAFF had no detectable impact on NK cell–mediated control of tumor burden in the lungs.

The B16‐F1 melanoma cell line leads to a less aggressive cancer than B16‐F10 and is therefore useful for studying primary tumor growth.^37^ B16‐F1 melanoma cells injected subcutaneously into the flank of each mouse formed a visible tumor at the injection site. Each curve in Figure 4b represents the tumor growth rate of an individual mouse. We did not observe any differences in the rates of tumor growth in BAFF^−/−^ or BAFF Tg mice relative to the WT controls (Figure 4b). We also examined cumulative survival using this tumor model. The ethical endpoint was set at a tumor size of 0.5 cm^3^. We found that the survival rates of the BAFF^−/−^ and BAFF Tg mice were statistically indistinguishable from those of the WT controls (Figure 4b). Thus, we conclude that modulating BAFF expression has no effect on this tumor model, which suggests that it is not required for NK cell–mediated control of tumor growth *in vivo*.

### NK cells do not express receptors that bind to BAFF

We showed that NK cell population size and maturation were affected in some mice lacking BAFF or its receptors. This raised the question as to whether this effect was caused by the loss of BAFF signaling in NK cells themselves or as an indirect consequence of impaired BAFF signaling in other cells, such as B cells. To address this issue, we evaluated the expression of BAFF‐binding receptors in NK cells by flow cytometry. In the absence of a reliable antibody to BCMA protein, we also examined messenger RNA expression of the receptors to BAFF in NK cells by real‐time quantitative PCR (RT‐qPCR).

**Figure 5:**
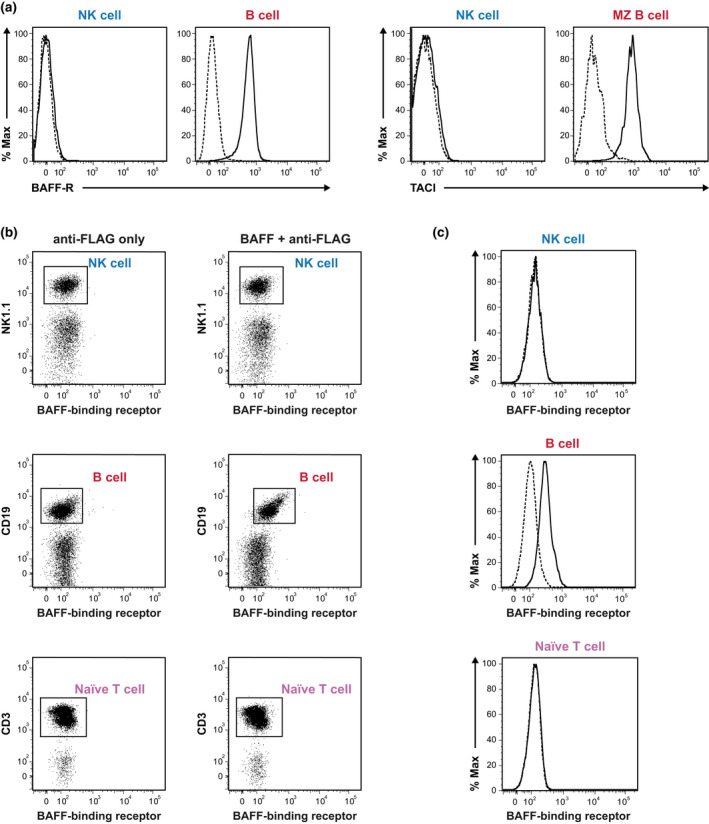
Flow cytometry analysis of receptor expression on resting NK cells from WT mice. **(a)** Surface expression of BAFF‐R and TACI on splenic NK cells. *Left*: solid (WT) and dotted (BAFF‐R^−/−^) lines represent BAFF‐R expression on NK cells from WT and BAFF‐R^−/−^ mice, respectively, with B cells as positive controls. *Right*: solid and dotted lines represent the results of staining NK cells from WT mice with an anti‐TACI antibody and fluorescence minus one control, respectively. Splenic MZ B cells (CD19^+^IgM^high^CD21/35^high^CD23^−^CD93^−^) were used as positive controls. The data shown are representative of three experiments that yielded similar results. **(b)** Splenocytes from WT mice were incubated either with or without FLAG‐tagged mouse recombinant BAFF followed by anti‐FLAG M2 FITC secondary antibody. CD19^+^ B cells and naïve T cells (CD19^−^CD3^+^CD44^low^CD62L^hi^) served as positive and negative controls, respectively. NK cells were identified as CD19^−^CD3^−^NK1.1^+^. **(c)** Histograms document the binding of recombinant BAFF to NK, B and naïve T cells. Dotted and solid lines denote cells detected only with anti‐FLAG and cells detected with recombinant BAFF together with anti‐FLAG, respectively. The data shown are representative of two independent experiments with 2 mice per group (*n* = 4 mice). BAFF, B‐cell–activating factor; BAFF‐R, B‐cell–activating factor receptor; FITC, fluorescein isothiocyanate; MZ, marginal zone; NK, natural killer (cells); TACI, transmembrane activator and calcium modulator and cyclophilin ligand interactor; WT, wild type.

**Figure 6:**
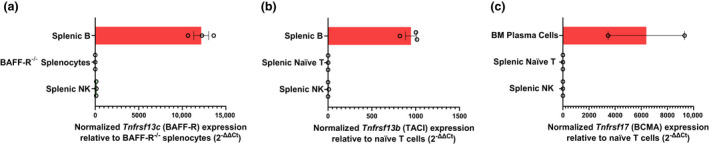
RT‐qPCR analysis of BAFF‐R, TACI and BCMA expression in resting NK cells from WT mice. The expression of **(a)** *Tnfrsf13c* (BAFF‐R), **(b)** *Tnfrsf13b* (TACI) and **(c)** *Tnfrsf17* (BCMA). NK cells were isolated to over 96% purity from the spleens of three WT mice through fluorescence‐activated cell sorting. B cells were used as positive controls for BAFF‐R and TACI, and BM plasma cells were used as positive controls for BCMA. Splenocytes from BAFF‐R^−/−^ mice were used as negative controls for BAFF‐R, and naïve T cells were used as negative controls for TACI and BCMA. Total RNA was purified from these cells, then reverse transcribed into cDNA. *Tnfrsf13c, Tnfsf13b, Tnfrsf17* and the *Hprt* internal control were then quantified in triplicate for each mouse. The fold change of each gene of interest was calculated as 2^–ΔΔCt^ (Ct: cycle threshold). ΔΔCt = (Ct gene of interest – Ct *Hprt*) of each cell type – (Ct gene of interest – Ct *Hprt*) of negative control. Samples that generated “undetermined” Ct values were assigned a value of 40 (the limit of detection). Each data point represents the mean ± standard error of the mean for an individual biological replicate. The data shown are representative of three biological replicates, with the exception of BM plasma, which represents two biological replicates. BAFF, B‐cell–activating factor; BAFF‐R, B‐cell–activating factor receptor; BCMA, B‐cell maturation antigen; BM, bone marrow; cDNA, complementary DNA; NK, natural killer (cells); RT‐qPCR, real‐time quantitative PCR; TACI, transmembrane activator and calcium modulator and cyclophilin ligand interactor; WT, wild type.

BAFF‐R and TACI protein expression were both undetected on the surface of splenic NK cells, as compared with their positive controls, B cells and marginal zone B cells, respectively (Figure 5a). While messenger RNAs encoding BAFF‐R, TACI and BCMA were observed in B cells, they were either undetectable or expressed at comparable levels to negative controls in splenic NK cells (Figure 6a–c). No signal was detected in the no–reverse transcriptase control, thus confirming the absence of genomic DNA contamination. RNA sequencing analysis of the Immunological Genome Project (ImmGen) database confirmed our observations (Supplementary figure 2).

To test the possibility that NK cells express other uncharacterized receptors that bind to BAFF, we incubated splenocytes with FLAG‐tagged BAFF followed by a fluorescein isothiocyanate (FITC)–tagged secondary anti‐FLAG M2 antibody. As expected, the B cells clearly interacted with FLAG‐tagged BAFF in this assay (Figure 5b, c). As with the naïve T cells that served as a negative control (Figure 5b, c), we did not detect any BAFF binding on the NK cells (Figure 5b, c). Collectively, our results demonstrate that BAFF was not capable of directly binding to the NK cells. Therefore, differences in NK cell numbers and maturation seen in mice deficient in BAFF or its receptors are an indirect effect linked to the alteration of the cellular environment.

### Preserved expression pattern of BAFF‐binding receptors in the lupus model (BAFF Tg mice)

**Figure 7:**
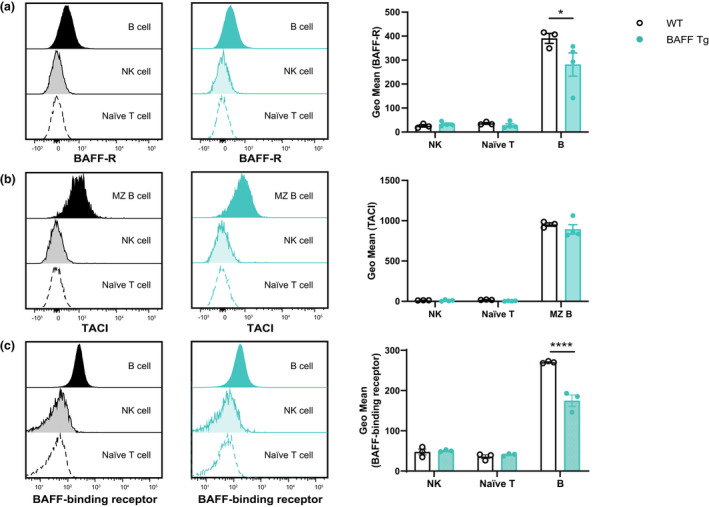
Comparison of BAFF‐binding receptor expression between BAFF Tg and WT littermate controls. The surface expression of **(a)** BAFF‐R and **(b)** TACI on splenic NK cells from WT mice as compared with BAFF Tg mice. **(c)** Splenocytes were incubated with FLAG‐tagged BAFF followed by anti‐FLAG antibody to detect the presence of receptors that interact with BAFF. Naïve T cells served as negative controls, whereas B cells served as positive controls. Staggered histograms represent the fluorescence intensities of staining of NK, naïve T and B cells from WT (left) and BAFF Tg (right) mice with anti‐BAFF‐R, anti‐TACI and anti‐FLAG antibodies. Data shown in the bar graphs are mean ± standard error of the mean from BAFF Tg mice (*n* = 3 or 4) and WT littermate controls (*n* = 3). Data are representative of two or three independent experiments (*n* = 6–10) yielding similar results. *P ≤ 0.05, ****P ≤ 0.0001. BAFF, B‐cell–activating factor; BAFF‐R, BAFF receptor; MZ, marginal zone; NK, natural killer (cells); TACI, transmembrane activator and calcium modulator and cyclophilin ligand interactor; Tg, transgenic; WT, wild type.

Although we established that BAFF‐binding receptors were not expressed at steady state, characterizing the expression of these receptors in a disease setting was important; therefore, we used the lupus model of BAFF Tg mice. The expression levels of BAFF‐R (Figure 7a) and TACI (Figure 7b) on NK cells from BAFF Tg mice were indistinguishable from those of WT littermate controls. By utilizing FLAG‐tagged recombinant BAFF and fluorescently labeled anti‐FLAG antibody, we confirmed that the expression of BAFF‐binding receptors was not upregulated in our lupus model (Figure 7c). This observation enabled us to exclude the link between BAFF and NK cells in lupus.

## DISCUSSION

NK cells play essential roles in suppressing cancer and infection, and may also control autoreactive cells.^18^ Considerable research has focused on the role of BAFF in B‐cell responses, but the impact of BAFF on innate immune responses has not been studied as extensively. Monocytes and macrophages respond to BAFF *via* interactions with TACI^38^, ^39^ and previous studies have suggested that BAFF might have an indirect role in promoting increased NK cell cytotoxicity *via* the production of IL‐2 and IFNγ by CD4^+^ T cells.^25^, ^26^ Here, our work using various genetically modified (GM) mice of the BAFF system clarified the role of BAFF and its receptors in NK cell homeostasis and function.

We showed that the numbers of NK cells were altered in B‐cell–deficient BAFF^−/−^ and BAFF‐R^−/−^ mice. However, BAFF is dispensable for NK cell function. Although NK cells produce BAFF,^20^, ^24^ they do not express receptors for BAFF. As NK cells do not express BAFF‐R, TACI or BCMA, the effects on NK cell numbers and maturation in BAFF^−/−^ and BAFF‐R^−/−^ mice are indirect. Abnormal NK cell numbers in the spleens of BAFF^−/−^ and BAFF‐R^−/−^ mice may be due to the absence of mature B cells. In support of this hypothesis, the survival of splenic NK cells was compromised in B‐cell–deficient μMT mice.^40^ The number of NK cells in the BM and liver in our study was within normal ranges, so our finding might be attributed specifically to B‐cell–mediated support in the generation of a normal splenic architecture. Mature B cells are required for stromal cell development and dendritic cell accumulation.^41^, ^42^ Stromal cells and dendritic cells are important sources of IL‐15, which is a key cytokine involved in regulating NK cell maturation, survival and function.^43^ In addition to secreting IL‐15, dendritic cells express IL‐15Rα, which binds to soluble IL‐15. This IL‐15–IL‐15Rα complex is trans‐presented to NK cells, which express IL‐2/15Rβ and γc heterodimers.^43^, ^44^ The alteration of splenic architecture which impedes efficient contact between cells is likely to affect the trans‐presentation of IL‐15. Although NK cell survival is dependent on dendritic cell–driven IL‐15 trans‐presentation, soluble IL‐15 is sufficient to promote NK cell maturation in the spleen.^44^ This may explain why B‐cell deficiency in the spleen greatly reduced NK cell numbers in BAFF^−/−^ and BAFF‐R^−/−^ mice, but only marginally impaired NK cell maturation in BAFF‐R^−/−^ mice. Although IL‐15 has been identified as a positive regulator of NK cell function,^45^ our findings are consistent with previous work^40^ documenting unaltered NK cell activity in B‐cell–deficient mice.

We observed expanded NK populations in the BM of TACI^−/−^ and BCMA^−/−^ mice. While the mechanisms underlying this expansion remain to be determined, it may be attributed to the interactions between TACI and BCMA and the BAFF homolog APRIL.^1^ We also showed that TACI deletion augmented NK cell development and survival in the spleen. This change is associated with extrinsic factors, as we demonstrated that NK cells do not express TACI.

Interestingly, reduced NK numbers have been reported in patients diagnosed with SLE.^27^, ^46^ Although BAFF Tg mice have been used as a murine model of SLE, our phenotypic data revealed that their NK cell population sizes were not significantly different from those of their WT littermate controls. These discrepancies reflect the heterogeneous nature of SLE that prevents direct comparison. Indeed, SLE is a polygenic disorder, whereas autoimmunity in BAFF Tg mice results from the overexpression of a single gene.^14^, ^47^ Furthermore, only 20–67% of patients with SLE display abnormally high levels of BAFF.^48^ Our results imply that NK cell deficiencies associated with SLE may result from dysregulation of other genes or factors. However, it is important to note that a loss of NK cells as a result of belimumab‐mediated loss of B cells may have consequences on tumor surveillance in patients treated this way, a risk that has already been identified as a possible adverse event with this treatment.^49^

We did not observe any correlation between BAFF and NK cell activity in our study. However, previous *in vitro* and *in vivo* studies documented correlations between BAFF levels and NK cell cytotoxicity.^25^, ^26^ For example, NK cell–mediated cytotoxicity was enhanced in both *in vivo* and *in vitro* settings in response to challenges with increasing concentrations of BAFF.^25^, ^26^ However, the impact of BAFF on NK cells may be indirect and mediated by interactions between BAFF and BAFF‐R expressed by activated T cells. Others have reported that BAFF alone had no impact on NK cell activity *in vitro*, and demonstrated that increased NK cell activity was observed when T cells were present in the cultures.^26^ The results from our *ex vivo* and *in vivo* experiments similarly indicate that NK cells from BAFF Tg mice displayed no enhanced capacity to lyse tumor cells.

Lastly, we found that genetic modification of the BAFF system had no impact on the formation of B16‐F10 lung melanoma nodules. The B16‐F10 mouse melanoma model has been widely used to assess NK cell activity *in vivo*. As the B16‐F10 melanoma cells express low levels of major histocompatibility complex class I, the rejection of tumor nodules in the lungs is not dependent on the actions of CD8^+^ cytotoxic T cells.^35^, ^36^ Our results suggest that BAFF is not required to support NK cell effector functions *in vivo*. The lack of any substantial change in the lung tumor burden in our study shows that the lung NK cell population in these GM mouse strains can perform effector functions similar to WT NK cells.

In summary, our study provides the first detailed investigation of the role of BAFF in supporting NK cell numbers and function. We found that dysregulated BAFF signaling had an impact on NK cell numbers in both the BM and the spleen. However, we determined that these NK cells do not express BAFF‐binding receptors; therefore, the effects of BAFF on NK populations are indirect. Rather, evidence from other studies suggests that an indirect contribution of B cells in the splenic environment may be required for NK populations in the spleen. Our *ex vivo* and *in vivo* experiments additionally ruled out BAFF having any impact on the functionality of NK cells; NK cells from mice lacking BAFF maintained cytotoxicity and IFNγ production at levels indistinguishable from WT mice. Considering the use of BAFF inhibitors in the clinic,^16^, ^17^ it is critical to carefully evaluate the impact of BAFF inhibition on various immune cell populations to better anticipate any direct or indirect side effects of removing BAFF, which we revealed in this study to be an indirect consequence on NK populations. Our study also demonstrates that the phenotype, function and BAFF‐binding receptor expression of NK cells remain unaltered in the BAFF Tg mouse model of lupus, which eliminates the possibility of any direct contributions of a BAFF–NK cell axis to the pathogenesis of lupus.

## METHODS

### Ethics statement

All experiments were performed in accordance with the Australian Code of Practice for the Care and Use of Animals for Scientific Purposes. The Alfred Medical Research and Education Precinct Animal Ethics Committee (ethics number: E/1365/2013/M) and the University of Melbourne Animal Ethics Committee (ethics number: 1513750) approved all experiments.

### Mice

The animals were bred and housed in specific pathogen‐free facilities at Alfred Medical Research and Education Precinct and the Doherty Institute. The BAFF Tg [B6.DBA‐Tg (Tnfsf13b)^tm1Fma^],^14^ BAFF^−/−^ [B6.129S2‐Tnfsf13b^tm1Msc^/J],^50^ BAFF‐R^−/−^ [B6(Cg)‐Tnfrsf13c^tm1Mass^/J],^2^ TACI^−/−^ [B6.129S2‐Tnfrsf13b^tm1Cmac^/J]^6^ and BCMA^−/−^ [B6.129S2‐Tnfrsf17^tm1Msc^/J]^50^ mice had been backcrossed to C57BL/6J mice for at least ten generations. All mice within each experiment were matched for both age and gender. The BAFF Tg mice were maintained as heterozygous for the transgene by backcrossing to C57BL/6J mice.

### Organ and tissue collection

The mice were killed in CO_2_ chambers and dissected for organ collection. To collect BM cells, the femurs were flushed with 5 mL of fluorescence‐activated cell sorting (FACS) buffer (phosphate‐buffered saline containing 2% fetal calf serum and 2 mM ethylenediaminetetraacetic acid). The livers were perfused with FACS buffer through the hepatic portal vein. The bile ducts were removed before the livers were placed in FACS buffer. The spleens were also excised and placed in FACS buffer.

### Flow cytometry

Single‐cell suspensions of BM, liver and spleen were prepared according to standard protocols. Liver mononuclear cells were separated from hepatocytes by centrifugation at 800 *g* in a 33% Percoll gradient. The cell suspensions were subjected to red blood cell lysis before being stained with specific antibodies. Cell concentrations in each suspension were adjusted to 1–4 × 10^6^ viable cells per well in 96‐well V‐bottomed plates. Counting beads (10 000) were also added to each well. Antibody panels used to detect specific immune cell populations and receptor expression included the following:

1. antibodies used to identify NK cells (anti‐CD3 APC, anti‐CD27 PE, anti‐NK1.1 PE‐Cy7, anti‐CD11b FITC and anti‐CD19 PerCP‐Cy5.5);
2. antibodies used to identify previously characterized NK cell receptors (anti‐CD19 PerCP‐Cy5.5, anti‐TCRβ V450, anti‐BAFF‐R PE or anti‐TACI PE and anti‐NK1.1 PE‐Cy7); B cells were used as positive controls and naïve T cells were used as negative controls for receptor expression;
3. antibodies used to identify B cells (anti‐IgM FITC, anti‐CD19 PerCP‐Cy5.5, anti‐CD93 APC, anti‐CD21/35 eFluor450, anti‐BAFF‐R PE or anti‐TACI PE and anti‐CD23 PE‐Cy7);
4. antibodies used to identify naïve T cells (anti‐CD62L FITC, anti‐CD44 APC, anti‐TCRβ V450 and anti‐BAFF‐R or anti‐TACI PE);
5. antibodies and reagents used to identify potential BAFF‐binding receptors (anti‐CD19 PerCP‐Cy5.5, anti‐CD44 APC, anti‐CD3 V450, anti‐CD62L PE and anti‐NK1.1 PE‐Cy7). FLAG‐tagged mouse recombinant BAFF (1 μg mL^−1^; AdipoGen Life Sciences, San Diego, CA, USA) and anti‐FLAG M2‐FITC (Sigma‐Aldrich, St. Louis, MO, USA) were used to detect secondary binding.

Information on all antibodies used for flow cytometric applications is included in Supplementary table 2. Nonspecific binding was blocked in all samples with anti‐CD16/32, and the Zombie Aqua Fixable Viability Kit (BioLegend, San Diego, CA, USA) was used to eliminate dead cells. Cells were fixed and permeabilized for intracellular staining with the Foxp3 Transcription Factor Staining Buffer Set (eBioscience, San Diego, CA, USA) followed by anti‐granzyme B AF647 staining. For experiments using mouse anti‐FLAG M2‐FITC antibody, an additional round of incubation with anti‐CD16/32 was conducted before primary and secondary staining to reduce nonspecific binding. Data were acquired using LSR Fortessa with FACSDiva software (BD Biosciences, Franklin Lakes, NJ, USA) and were analyzed using FlowJo software (BD Biosciences, Franklin Lakes, NJ, USA).

### Chromium (^51^Cr) release assay

The spleens were collected and processed into single‐cell suspensions. NK cells were isolated from splenocytes to >90% purity, starting with cells from two pooled spleens, using the EasySep Mouse NK Cell Isolation Kit (Stemcell Technologies, Vancouver, CA) following the manufacturer's instructions. Target YAC‐1 cells were labeled with 100 μCi of Chromium‐51 (^51^Cr; Perkin Elmer, Waltham, MA, USA) for 75 min. A total of 10 000 labeled target cells were added to the wells of V‐bottomed plates, followed by effector NK cells at various effector‐to‐target ratios. Spontaneous ^51^Cr release was determined in YAC‐1 cultures (six wells) incubated in the medium alone. Hydrochloric acid (1 M) was added to three wells to maximize ^51^Cr release. After 4 h of incubation at 37°C and 5% CO_2_, supernatants were collected and ^51^Cr release was measured on the Wallac Wizard gamma Counter (Perkin Elmer, Waltham, MA, USA). We determined the percentage of labeled target cells that were lysed specifically by NK cells with the following formula:

$$\text{Specific lysis}=\left[\left(\text{experimental release}–\text{spontaneous release}\right)/\left(\text{maximum release}–\text{spontaneous release}\right)\right]\times 100.$$

### Tumor cell lines

The B16‐F1 and B16‐F10 melanoma cell lines were obtained from the American Type Culture Collection (Manassas, VA, USA) and were cultured at 37°C in a 5% CO_2_ incubator. Cells were harvested at a confluence of ≤ 70% (for B16‐F1 cells) or ≤ 50% (for B16‐F10 cells) using the TrypLE Express dissociation enzyme (Thermo Fisher Scientific, Waltham, MA, USA). The cells were passaged six times to generate enough cells to prepare frozen stocks for future use. Cells from the final passage (P6) were used in all experiments. Two independent cell cultures from P6 were passaged twice in antibiotic‐free medium and were screened for pathogens (Cerberus Sciences, Scoresby, VIC, Australia). All cells injected into the mice were documented free of mycoplasma contamination.

### The B16 melanoma tumor model

We shaved the right lateral flanks of the mice before subcutaneously injecting 0.5 × 10^5^ melanoma cells suspended in 100 μL of 1× Hank's balanced salt solution (Thermo Fisher Scientific, Waltham, MA, USA). The mice were monitored, and the tumor sizes were measured daily. The mice were killed 15 days after palpable tumors were detected or when the tumor size exceeded 500 mm^3^. Tumor volumes were determined based on measurements of their largest (a) and smallest (b) diameters using the following equation:

$$\text{Tumor volume}=\left(b^{2}\times a\right)/2.$$

### The B16 melanoma pulmonary metastasis model

The mice were placed under a heat lamp or heat pad to dilate their tail veins. A temperature probe was placed in the box to ensure that the mice did not overheat. After approximately 10 min, each mouse was placed in a restraint to facilitate the intravenous injection of 2 × 10^5^ B16‐F10 melanoma cells suspended in 100 μL of 1× Hank's balanced salt solution. Injections were performed using 29½ G insulin needles (Terumo, Shibuya City, Tokyo, Japan). Fifteen days after the melanoma cell injection, the mice were killed and lungs with visible tumor nodules were collected, washed with 1× phosphate‐buffered saline and fixed in Bouin's Fixative (Sigma‐Aldrich, St. Louis, MO, USA). The tumor nodules in each lung were counted under a dissection microscope.

### IFNγ production by splenic NK cells

NK cells were enriched using magnetic cell isolation methods (Miltenyi Biotec, Bergisch Gladbach, North Rhine‐Westphalia, Germany) that depleted CD19^+^ and CD3^+^ cells from the splenocyte pool. NK cells (CD19^−^CD3^−^NK1.1^+^) were then isolated to over 95% purity by FACS on an FACS Influx or an FACS Aria (BD Biosciences, Franklin Lakes, NJ, USA) cell sorter. Purified NK cells were resuspended to a concentration of 10^6^ cells mL^−1^ and cultured with or without recombinant human IL‐12 (1 ng mL^−1^; mouse cross‐reactive) and mouse IL‐18 (5 ng mL^−1^). Supernatants were collected after 6 h. IFNγ concentrations were measured using the LEGENDplex bead‐based immunoassay (BioLegend, San Diego, CA, USA) performed according to the manufacturer's instructions, with the exception that we used half the recommended volumes.

### Quantitative reverse transcription PCR

The purity of all immune cells used for RT‐qPCR was greater than 96%. RNA was extracted from various cell types using RNeasy Mini kit (Qiagen, Hilden, North Rhine‐Westphalia, Germany) according to manufacturer's instructions. Complementary DNA was synthesized using a standardized concentration of RNA and the High‐Capacity RNA‐to‐cDNA Mastermix (Applied Biosystems, Waltham, MA, USA). Each sample contained 11 μL of the Mastermix with or without reverse transcriptase and 9 μL of RNA templates diluted in diethyl pyrocarbonate (DPEC)‐treated water. RT‐qPCRs were carried out in a final volume of 12.5 μL containing (i) TaqMan Universal Mastermix without uracil‐N‐glycosylase (Applied Biosystems, Waltham, MA, USA), (ii) gene‐specific TaqMan primer/probe sets (Applied Biosystems, Waltham, MA, USA), (iii) complementary DNA and (iv) water. TaqMan primer/probe sets targeting *Hprt* (Mm03024075_m1), *Tnfrsf13c* (BAFF‐R; Mm00840578_g1), *Tnfrsf13b* (TACI; Mm00840182_m1) and *Tnfrsf17* (BCMA; Mm00495683_m1) were obtained from Applied Biosystems (Waltham, MA, USA). All primers were designed to span exon junctions and therefore to maximize specificity for complementary DNA targets. RT‐qPCR assays were performed using an Applied Biosystems 7900HT RT‐qPCR thermocycler. B cells (CD19^+^) were used as positive controls for the expression of BAFF‐R and TACI. BM plasma cells (B220^low^CD138^high^) were used as a positive control for the expression of BCMA. Splenocytes from BAFF‐R^−/−^ mice were used as the negative control for BAFF‐R; naïve T cells (CD3^+^CD44^low^CD62L^+^) were the negative control for TACI and BCMA.

### Statistical analysis

All statistical analysis and figure design were performed using GraphPad Prism 9 (GraphPad Software, San Diego, CA, USA). Prior to analysis, we tested each data set for a normal distribution using the Shapiro–Wilk test (for fewer than eight values) and the D'Agostino–Pearson test (for more than eight values). Welch's or Brown–Forsythe tests were used to test for unequal variances. When the data were collected for one independent variable, we used Student's *t*‐tests for comparisons between two groups of normally distributed data, and one‐way ANOVA for comparisons between more than two groups of normally distributed data. ANOVA was followed by Dunnett's (for data with equal variance) or Dunnett's T3 (for data with unequal variance) multiple comparison tests. Non‐normally distributed data were analyzed with the Mann–Whitney *U* test for comparison between two groups of data, or the Kruskal–Wallis test with Dunn's *post hoc* test for comparisons between more than two groups of data. When the data were collected for two independent variables, significance was determined with two‐way ANOVA, followed by Šídák's multiple comparisons *post hoc* test. The results were considered statistically significant if *P‐*values ≤ 0.05.

## AUTHOR CONTRIBUTIONS

**Pin Shie Quah:** Conceptualization; data curation; formal analysis; investigation; methodology; visualization; writing – original draft; writing – review and editing. **Vivien Sutton:** Formal analysis; investigation; methodology; writing – review and editing. **Eden Whitlock:** Investigation; writing – review and editing. **William A Figgett:** Conceptualization; methodology; writing – review and editing. **Daniel M Andrews:** Conceptualization; methodology; resources; supervision; validation; writing – review and editing. **Kirsten A Fairfax:** Conceptualization; formal analysis; methodology; project administration; supervision; validation; writing – review and editing. **Fabienne Mackay:** Conceptualization; funding acquisition; methodology; resources; supervision; validation; writing – review and editing.

## CONFLICT OF INTEREST

The authors declare no conflict of interest.

## Data Availability

The data and materials that support the findings of this study are available in article supplementary information and source data are available upon reasonable requests from the authors.
