# Supplementary material for: The effects of B‐cell–activating factor on the population size, maturation and function of murine natural killer cells
Source: Immunol Cell Biol. 2022 Oct 8;100(10):761–76. doi: 10.1111/imcb.12585 (PMC9828838; doi:10.1111/imcb.12585)
Supplement: Supplementary file 1 [file IMCB-100-761-s001.pdf]

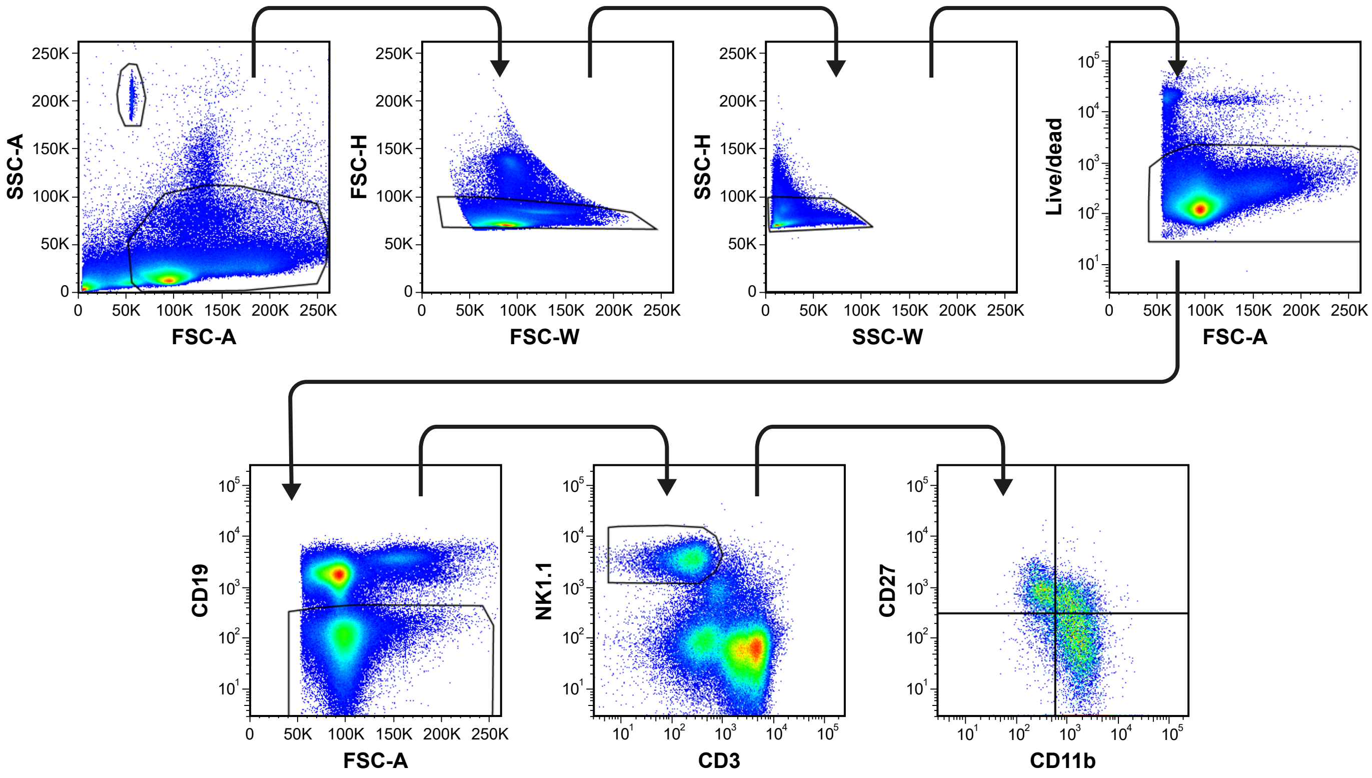

Supplementary figure 1. Gating strategy used to identify NK cells in mouse spleens. Single lymphocytic cells were initially identified based on their characteristic forward scatter (FSC) and side scatter (SSC) properties. Zombie^{TM} Aqua Fixable Viability Kit was used to exclude dead cells. Cells within the CD19^{-} gate were identified to exclude lineage cells. NK cells were identified as the CD3^{-} NK1.1^{+} population, which was then divided into distinct maturation stages based on the expression of CD11b and CD27.

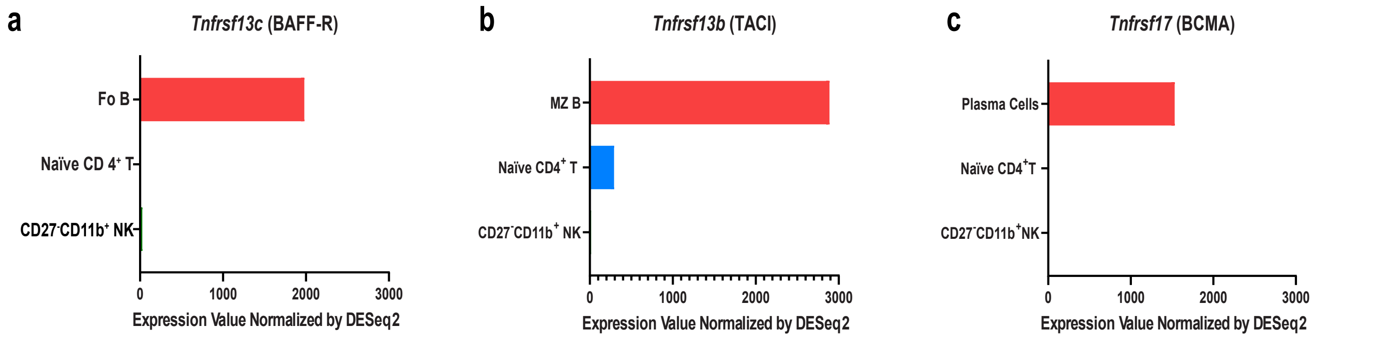

Supplementary figure 2. RNA sequencing (RNAseq) analysis of **(a)** *Tnfrsf13c* (BAFF-R), **(b)** *Tnfrsf13b* (TACI) and **(c)** *Tnfrsf17* (BCMA) in splenic CD27$^-$CD11b$^+$NK cells, naïve T cells and B cells from 6 weeks old C57BL/6J mice (*n* = 1–3). B cells consists of follicular (Fo) B cells, marginal zone (MZ) B cells and plasma cells. The gene expression values are derived from the Immunological Genome Project (ImmGen) database. Cell types are colour-coded: B cells (red), naïve T cells (blue) and natural killer (NK) cells (green).

Supplementary table 1. Genetically-modified mice of the BAFF system

Genotype	Phenotype	Genetic modification†	Reference
BAFF$^{-/-}$	Mice exhibit impaired B cell survival and maturation.	Exons 1 and 2 (732 bp) that include the first 134 residues of the coding sequence were replaced with a human CD2 reporter gene fused to a neomycin-resistance cassette	Schiemann, B, Gommerman, JL, Vora, K, *et al.* An essential role for BAFF in the normal development of B cells through a BCMA-independent pathway. *Science*. 2001; **293**: 2111-2114.
BAFF-R$^{-/-}$	Mice exhibit impaired B cell survival and maturation.	Cre-recombinase-mediated excision of a floxed region containing exons 3 and 4. The excised region encoded the entire extracellular and transmembrane domains and part of the cytoplasmic domain of this receptor.	Sasaki, Y, Casola, S, Kutok, J, *et al.* TNF family member B cell-activating factor (BAFF) receptor-dependent and -independent roles for BAFF in B cell physiology. *J Immunol*. 2004; **173**: 2245-2252.
TACI$^{-/-}$	Mice exhibit increased numbers of peripheral	A tail-less human CD2 reporter cDNA and PGK-	Von Bülow GU, Van Deursen JM, Bram RJ. Regulation of the T-independent humoral response by TACI. *Immunity* 2001; **14**: 573-582.

	B cells, decreased serum IgA levels, and impaired responses to T cell-independent antigens, most notably those that are Type II.	neo cassette replaced 6.12 kb immediately downstream of the start ATG codon. This resulted in the insertion of stop codons in all potential reading frames downstream of the inserted reporter.	
BCMA$^{-/-}$	Mice have reduced number of long-lived bone marrow plasma cells	A region of the gene containing the translation initiation codon and 1.3 kb of downstream sequences were replaced with a human CD2 reporter gene fused to a neomycin resistance cassette. The mutation deletes the first 87 residues, which includes a putative transmembrane domain.	Schiemann, B, Gommerman, JL, Vora, K, *et al.* An essential role for BAFF in the normal development of B cells through a BCMA-independent pathway. *Science*. 2001; **293**: 2111-2114.
BAFF Tg	Mice overproduce BAFF and have increased numbers of	Full-length murine Tnfsf13b (BAFF) was expressed under the control of the human	Mackay, F, Woodcock, SA, Lawton, P, Ambrose C, Baetscher M, Schneider P. Mice transgenic for BAFF develop lymphocytic disorders

	peripheral B cells. These mice develop symptoms of an autoimmune disorder from approximately 8 weeks of age.	liver-specific alpha-1-antitrypsin promoter with an apolipoprotein E enhancer.	along with autoimmune manifestations. *J Exp Med*. 1999; **190**: 1697-1710.

† The information included under genetic modification was extracted from the Mouse Genome Database (MGD) at the Mouse Genome Informatics website, The Jackson Laboratory, Bar Harbor, Maine. World Wide Web (URL: http://www.informatics.jax.org).

Supplementary table 2. Antibodies used in this experiment.

Target antigen	Fluorochrome/conjugate	Clone	Isotype	Manufacturer
BAFF-R	PE	TH22-E16	Rat IgG1, κ	Biolegend
CD3	APC<br>V450	145-2C11<br>17A2	Armenian Hamster IgG<br>Rat IgG2b, κ	Biolegend<br>BD
CD11b	FITC	M1/70	Rat IgG2b, κ	BD
CD16/32 (Fc block)	-	2.4G2	Rat IgG2b, κ	BD
CD19	PerCP-Cy5.5	1D3	Rat IgG2a, κ	BD
CD21/35	eFluor450	eBio4E3	Rat IgG2a, λ	eBioscience
CD23	PE-Cy7	B3B4	Rat IgG2a, κ	eBioscience
CD27	PE	LG.3A10	Armenian Hamster IgG1, κ	BD
CD44	APC	IM7	Rat IgG2b, κ	eBioscience
CD62L	FITC<br>PE	MEL14<br>MEL14	Rat IgG2a, κ<br>Rat IgG2a, κ	BD<br>BD
CD93	APC	AA4.1	Rat IgG2b, κ	eBioscience
Granzyme B	AF647	GB11	Mouse (BALB/c) IgG1, κ	BD
IgM	FITC	II/41	Rat IgG2a, κ	BD
NK1.1	PE-Cy7	PK136	Mouse (C3H x BALB/c) IgG2a, κ	BD
TACI	PE	8F10	Rat IgG2a	Biolegend
TCRβ	V450	H57-597	Mouse IgG2, λ1	BD
